# Distribution and Risk Assessment of Copper Content in Soil and Tea of Tieguanyin Plantations in Anxi County, China

**DOI:** 10.3390/toxics13121042

**Published:** 2025-11-30

**Authors:** Qiyu Zeng, Yuanyuan Zhan, Changwu Tao, Kaijun Feng, Jingya Zheng, Huogui Su, Yuede Wu

**Affiliations:** 1 College of Resources and Environment, Fujian Agriculture and Forestry University, Fuzhou 350002, China; fafuzhyzyy@163.com (Y.Z.); taocw779@163.com (C.T.); fengkaijun666@163.com (K.F.); 2 Fujian Forestry Prospect and Design Institute, Fuzhou 350005, China; 3 Anxi County Soil Fertilizer Technology Extension Station, Quanzhou 362400, China; zjy5905@126.com (J.Z.); 13505918807@163.com (H.S.); wuyuede1234@163.com (Y.W.)

**Keywords:** Tieguanyin tea, soil-tea system Cu content, spatial distribution, ecological risk assessment, human health risk assessment

## Abstract

Cu in tea leaves can be easily leached into the tea broth during brewing and ingested by humans; therefore, excessive accumulation of Cu in tea leaves may pose potential health risks. In this study, the relationship between soil Cu and Cu content in tea plantations was investigated by analyzing 106 surface soil samples (0–20 cm) and their corresponding tea samples from Anxi County. The distribution of Cu, ecological risk, and early warning indicators were analyzed in both tea and soil samples. Research indicates that soil Cu content is classified into five grades in accordance with the Specifications for Geochemical Evaluation of Land Quality: Grade I (>29 mg/kg, accounting for 7.55%), Grade II (24~29 mg/kg, accounting for 2.83%), Grade III (21~24 mg/kg, accounting for 3.77%), Grade IV (16~21 mg/kg, accounting for 8.49%), and Grade V (≤16 mg/kg, accounting for 77.36%). The mean soil Cu content varied with the underlying rock type, following the order: sedimentary rocks > metamorphic rocks > magmatic rocks. The spatial distribution of soil Cu content was higher in the north and lower in the southeast and northwest, whereas tea Cu content was higher in the southeast and northwest and lower in the central region. Soil-forming parent materials, traffic emissions, and agricultural activities were identified as the main sources of soil Cu, while tea Cu content was mainly affected by soil Cu and agricultural activities. Importantly, soil Cu posed no significant ecological risks, and all tea samples had Cu contents within safe limits without significant toxicity or health hazards. This study innovatively integrates Cu source identification, spatial analysis, and dual-dimensional (soil–tea) risk assessment in a typical tea-producing area, providing a targeted scientific basis for the precise monitoring and management of soil Cu in tea plantations and ensuring compliance of soil and tea products with national safety standards.

## 1. Introduction

Tea, one of the world’s major beverages, is a valuable natural drink with numerous health benefits. Historical records indicate that China has cultivated tea for over 2000 years, with extensive planting across the southern provinces. Increasing demand for tea has led to changes in tea production practices, which can affect not only soil physicochemical properties and tea quality but may also affect the migration, transformation, and accumulation of heavy metal elements in the soil–tea system [1]. Heavy metal accumulation in tea has long been a research focus in food safety and environmental science. Tea plantation ecosystems are often affected by multiple heavy metal pollutants, including Cu, Pb, Zn, Cd, Cr, and As, which originate from natural sources such as parent material weathering and anthropogenic activities like pesticide application, fertilizer use, and atmospheric deposition [2,3,4]. Among these metals, Pb and Zn are mainly associated with exogenous inputs such as traffic emissions and organic fertilizer application, while Cd and Cr are prone to accumulation in acidic soils and may pose chronic health risks through tea consumption [5,6]. Zn and Mn, as essential micronutrients for tea plants, participate in photosynthesis and enzyme activation, but excessive accumulation can also disrupt plant metabolism [7,8].

However, compared with other metals, Cu exhibits unique characteristics in the soil–tea system that make it a key research object. Tea leaves from trees grown in acidic and Cu-enriched soils tend to have higher Cu contents [3,9]. When tea is consumed, Cu from the tea broth enters the human body and can gradually accumulate [10]. As an essential trace element, copper plays a key role in metabolic processes such as enzyme catalysis and redox reactions [11]; however, excessive intake may pose a serious threat to human health. Studies have confirmed that abnormal accumulation of copper in the body is closely related to neuroinflammation and may increase the risk of neurodegenerative disorders such as Alzheimer’s disease [5].

Soil environmental conditions play a crucial role in determining tea quality. The soil-forming parent material, as the most direct material source, plays a decisive role in the total Cu content of the soil through its mineral composition, elemental abundance, and weathering characteristics [12]. Studies have shown that total Cu average contents rank as old weathered crust > sand shale > quartz sandstone. Soil particle composition also plays an important role in regulating the spatial distribution of Cu, with total Cu content strongly negatively correlated with sand grain content and strongly positively correlated with clay grain content [13]. Soil Cu content also has different thresholds under specific land-use types. In their study of rice cultivation in a typical carbonate rock area, Wang et al. [14] noted that maintaining soil Cu content within 6–84 mg/kg can effectively protect human health and reduce ecological risks. In tea plantations, soil Cu content must be lower than the national organic (natural) tea production and processing technology standards [15]. Bin et al. [16] found that copper content in tea garden soils exhibits a high-density, multi-concentric distribution pattern. This pronounced spatial variability is influenced by topography, elevation [17], and local microclimates. Anthropogenic activities in tea plantations also affect soil Cu content and tea quality. The widespread use of chemical fertilizers, herbicides, pesticides, and other chemical substances in tea plantations has resulted in the heavy metal contamination of both soil and tea to varying degrees; the long-term consumption of such tea can lead to chronic accumulation [2].

Soil serves as the primary reservoir of heavy metals for terrestrial plants, with metal migration from soil to plants occurring primarily through root uptake, followed by translocation to aboveground tissues—a process regulated by both soil properties and plant physiological characteristics [18,19]. For tea plants, which rely on acidic soils for optimal growth, the low-pH environment enhances the bioavailability of Cu^2+^ by reducing its adsorption to soil colloids, thereby promoting Cu uptake by tea roots [3,9]. Plant roots exhibit selective absorption of Cu—prioritizing available Cu fractions (e.g., DTPA-extractable Cu) over inert fractions, which explains the stronger correlation between plant Cu and soil available Cu compared to total soil Cu [18].

Copper is an essential micronutrient absorbed by plants through the root system, participating in various physiological and metabolic processes [7]. Cu deficiency can slow plant growth, cause deformation or whitening of sprouts, and curl leaf edges, ultimately reducing yield. Conversely, excessive Cu uptake can yellow plant tissues and inhibit Fe uptake and translocation by the root system. Thus, both insufficient and excessive plant Cu intakes pose risks [20]. Yemane et al. [21] reported that the Cu content in tea ranges from 0.73 to 19.15 mg/kg; however, the source of Cu in tea and its associated health risks were not evaluated. Health risk assessment models typically evaluate carcinogenic and noncarcinogenic risks through three exposure routes: inhalation, direct ingestion, and dermal contact [5]. Zhang et al. [3] demonstrated that Cu can be absorbed from the soil by tea tree roots and accumulated in tea leaves, indicating that soil is a major source of Cu in tea. Previous studies have found that long-term consumption of tea broth with high Cu content poses a carcinogenic risk [6]. However, the Cu content in Pu’er tea presents no carcinogenic risk to humans, and Cu levels in tea broth are lower than that in tea leaves [22,23], likely due to differences in steeping duration and frequency [24]. Therefore, assessing potential tea-related risks solely based on Cu content in tea broth is limited, whereas using Cu content in tea leaves provides a more reliable basis for evaluating such risks.

Anxi County is the largest tea-producing county in Fujian Province. Its Tieguanyin Tea Culture System has been recognized by the Food and Agriculture Organization of the United Nations (FAO) as a Globally Important Agricultural Heritage System (GIAHS), and its traditional tea-making techniques are also listed as an Intangible Cultural Heritage of Humanity by the United Nations Educational, Scientific and Cultural Organization (UNESCO). As a well-known tea-producing region in China, Anxi County has a unique geological background, with 54.2% of its area composed of volcanic rocks rich in Cu and other trace elements. Soils derived from these rocks are enriched in minerals and Cu, which promote the formation of tea polyphenols, amino acids, and other quality-related components. In this study, 106 representative tea plantations in Anxi County were selected to investigate the Cu content, spatial distribution patterns, and potential risks in both soils and leaves. The specific objectives were as follows: (1) clarify the distribution characteristics and key influencing factors of copper content in tea garden soils of Anxi County, filling a research gap on soil copper migration patterns under unique geological conditions; (2) elucidate the coupling mechanism between soil copper and tea leaf copper content, precisely tracing the primary sources of copper in tea leaves to provide scientific support for deciphering copper transfer pathways within the “geology–soil–tea leaf” system; and (3) conduct a comprehensive assessment of the potential ecological risks associated with copper in tea gardens and innovatively apply the RBCA model to evaluate health risks, quantitatively quantifying the copper-related health risks tea drinkers may face through tea consumption. This provides targeted technical support for quality safety management and sustainable development of the Anxi Tieguanyin tea industry.

## 2. Materials and Methods

### 2.1. Research Overview

**Figure 1:**
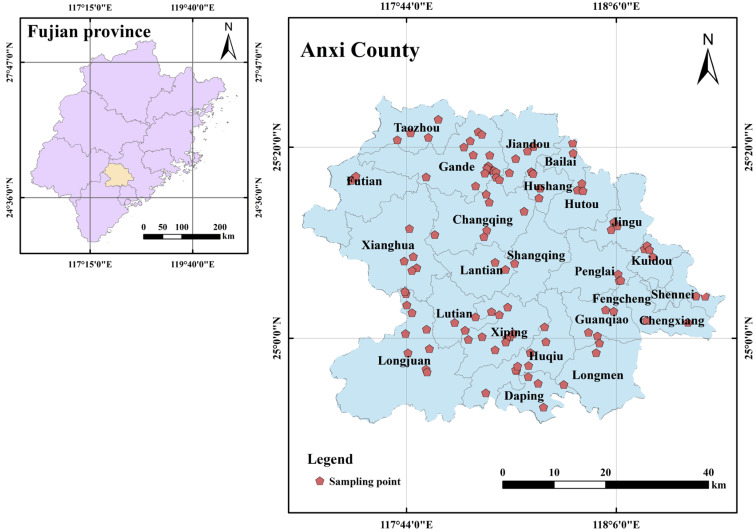
Study area location map.

Anxi County, the hometown of Chinese oolong tea and the birthplace of Tieguanyin, the world’s most famous tea, ranks first among China’s key tea-producing counties. The tea planting area exceeds 40,000 hm^2^, with a production of 62,000 t. The county is located southeast of Fujian Province and southwest of Quanzhou City between 24°50′–25°26′ N and 117°35′–118°17′ E (Figure 1). It borders Nan’an City to the east, Hua’an County to the west, Tong’an District to the south, Yongchun County to the north, Changtai County to the southwest, and Zhangping City to the northwest. The county lies within the central and southern subtropical climate zones, characterized by long sunshine periods and abundant heat, rainfall, and water resources. Average annual precipitation ranges from 1500 to 2000 mm, annual sunshine lasts about 1850 h, and the average annual temperature is 16–21.0 °C. The geology in Anxi County includes volcanic, intrusive, sedimentary, and metamorphic rock types. Located in the southeastern extension of the Daiyun Mountains, the terrain slopes from northwest to southeast [25]. The western region consists mostly of high mountains, majority approximately 600–800 m above sea level, with numerous peaks above 800 m. The eastern region is dominated by hills with elevations below 500 m, with most within 100–300 m. The terrain is low with gently undulating hills, relatively wide river valleys, and numerous bead-shaped intermountain valley basins. The soil in the territory has a zonal distribution pattern starting with brick-red soil in the southeast, transitioning to red soil, then yellow soil toward the northwest [1].

### 2.2. Sample Collection and Processing

In each township of Anxi County, representative tea plantations were selected as sampling sites based on similar management practices (single-variety Tieguanyin), tea age, slope orientation, and soil thickness. From areas near each selected plantation, 2–3 pieces of unweathered rocks that were consistent with the parent rock were collected. Surface debris were removed, and the rocks were placed in labeled self-sealing bags for preservation. These rocks served as the background reference for the rocks collected at the sampling sites. A portion of the reference rocks were ground and passed through a 200-mesh sieve, then stored in self-sealing bags for the determination of heavy metal contents. The exposed rocks in the study area are dominated by magmatic rocks, with sedimentary rocks and metamorphic rocks scattered sporadically. A total of 106 samples were collected, including 88 magmatic rock samples, 5 metamorphic rock samples, and 13 sedimentary rock samples.

Soil samples were collected from the 0–20 cm topsoil layer, from the drip line to the tree trunk, in the selected tea plantations, avoiding fertilization points. Sampling followed an S-shaped or plum-bloom pattern. Five representative soil samples were collected from each plantation and thoroughly mixed on a plastic sheet to eliminate impurities. From this mixed soil, 1 kg was taken according to the quadrature method; sampling numbers were recorded, and the soil was stored in bags for transport to the laboratory for analysis. Soil samples collected were cleaned of roots and other impurities, then placed in a dry, well-ventilated room to air-dry naturally. Subsequently, they were crushed and passed through a 2 mm sieve. Random subsamples from the sieved material were then ground and passed through a 0.074 mm sieve. The processed samples were labeled, placed in sample bags, and stored for testing.

Tea samples were randomly collected from the selected tea plantations at the standard of one bud and three leaves, with approximately 120 g per sample placed in envelopes. The sampling number was recorded, and the samples were quickly packed into foam boxes with ice bags to prevent oxidization. Samples were transported to the laboratory as soon as possible, where the thermosetting method used for fixation was conducted in an oven at 105 °C for 15 min. The oven temperature was then adjusted to 75 °C to dry the samples until a constant weight was achieved. The dried tea samples were ground, then placed in self-sealing bags with labels. The samples were stored in a refrigerator at −80 °C for preservation until analysis.

### 2.3. Experimental Methods

The detection methods for rock mineral elements were based on the national standard GB/T 14505-2010 [26], with determined indicators including Mn, Zn, Cu, Pb, etc. For soil available Cu, the diethylenetriaminepentaacetic acid extraction method was adopted, following Determination of Available Trace Elements in Soils—Part 2: Determination of Available Copper, Zinc, Iron, and Manganese in Soils by Atomic Absorption Spectrophotometry (NY/T 890-2004) [27]. Trace elements such as Mn, Zn, Cu, Ni, and Pb in the soil and Cu in the tea were determined using inductively coupled plasma mass spectrometry (ICP-MS 2000E, Jiangsu Tianrui Instruments Co., Ltd., Kunshan, China), according to the method DZ/T 0279-2016 [28]. The composition of soil particles was determined using a laser particle sizer (Model BT-9300ST, manufactured by Bettersize Instruments Ltd., Dangdong, China). Cu has a spatial distribution pattern similar to Zn, Ni, Pb, and Mn and shares the same source [29,30]; hence, Zn, Mn, and other metals were selected for the correlation analysis. The soil pH was determined using a pH meter assay (FE28-Standard pH Meter, Mettler-Toledo Instruments (Shanghai) Co., Ltd., Shanghai, China) based on the NY/T 1121.2-2006 [31] method. Organic matter content was determined using the acid–base titration method (NY/T 1121.2-2006) [31]. Data on manganese, lead, zinc, and iron content in rock and soil, along with soil pH, organic matter, and particle size distribution, are detailed in Supplementary Material S1. When determining the copper (Cu) content in soil and tea leaves, standard substances were added to ensure the accuracy and reliability of the measurement data. The detection limits were 0.49 mg/kg for total soil Cu, 0.005 mg/kg for available soil Cu, and 0.046 mg/kg for Cu in tea leaves, respectively. The quality assurance/quality control (QA/QC) procedures for all analytical testing are detailed in Supplementary Material S2.

### 2.4. Analytical Methods

#### 2.4.1. Soil Heavy Metal Health Risk Assessment Methods

The ecological risk evaluation method for heavy metals mainly focuses on analyzing soil contamination by metal elements. In this study, the Hakanson [32] potential ecological hazard index method was used to evaluate metal contamination. When applying this method, the toxicity and effectiveness of metals were fully considered, and the corresponding toxicity response coefficients were used to calculate the potential ecological hazard index (*E*) of individual HM elements. The grading of the ecological hazards of single-factor pollutants and the toxicity coefficients are shown in Supplementary Material S3. The calculation formulas are as follows:

(1) $$P=\frac{C}{S}$$

(2) E=T×P

*C*—is the measured concentration of polluted HMs;
*S*—is the background value determined by the standard in the study area;
*P*—is the pollution enrichment factor of HMs elements;
*T*—is the toxicity factor (Cu = 5);
*E*—is the potential ecological hazard index of HMs elements.

#### 2.4.2. Health Risk Assessment for Tea

The health risk assessment model established by the U.S. Environmental Protection Agency (USEPA) was used to evaluate the potential noncarcinogenic health risk caused by human intake of heavy metals, expressed as the target hazard quotient (*THQ*). A *THQ* < 1 indicates no significant noncarcinogenic risk, while the noncarcinogenic risk increases with an increase in *THQ* [33]. The estimated daily intake (*EDI*) of heavy metals is a key parameter in health risk assessment. Table 1 shows the calculated parameters of *EDI* and *THQ* which were determined as follows [34]:

(3) $$EDI=\frac{C\times E_{F}\times E_{D}\times F_{IR}}{W_{AB}\times T_{A}\times 1000}$$

(4) $$THQ=\frac{EDI}{RfD}$$

#### 2.4.3. Evaluation Criteria

This study adopted Chinese national and industry standards for evaluation, with clear division of applicable objects: soil Cu content was evaluated based on the Soil Environmental Quality Standards (GB 15618-2018) [39] and Environmental Conditions for Harmless Tea Origins (NY 5020-2001) [40], which specify the permissible limits of soil Cu in tea plantations (Supplementary Materials S4); and tea leaf Cu content was evaluated in accordance with the Green Food Tea (NY/T 288-2018) [41] and Organic Tea (NY 5196-2002) [42] standards, which define the hygiene limit indices for Cu in tea products (Supplementary Materials S5).

### 2.5. Data Processing

Frequency distribution histograms and scatter plots were generated using Excel 2010. SPSS 26 was employed to perform basic statistical analysis of data and conduct significance analysis based on data types. Origin 2018 was used for correlation analysis and heatmap creation. Canoco 5 software was adopted for principal component analysis (PCA) and corresponding graphing. After determining semivariogram parameters through fitting with GS^+^, ordinary kriging was applied for spatial interpolation in GIS 10.2, and high-resolution distribution maps were exported following classification via the Jenks natural breaks method and addition of map elements.

## 3. Results

### 3.1. Distribution Characteristics of Soil and Tea Cu Content

#### 3.1.1. Soil and Tea Cu Content Under Different Parent Rock Backgrounds

The parent rock serves as the basis for soil formation. Differences in the composition of mineral elements in various parent rocks are inevitably reflected in the mineral element content of the soils derived from them. Rocks are primarily classified into three major categories: sedimentary rocks, igneous rocks, and metamorphic rocks. Sedimentary rocks form through the transport, deposition, and lithification of loose sediments such as weathered parent material, exhibiting layered structures with visible ripple marks or fracture patterns on their surfaces. Igneous rocks are subdivided into intrusive and extrusive types, lacking layered structures. Intrusive rocks exhibit a fully crystalline coarse-grained texture, while extrusive rocks often display glassy or vesicular textures. Metamorphic rocks form through solid-state transformation of parent material under high temperature, pressure, and chemical fluid influence, exhibiting foliation or gneissic structures.

Normality tests (Shapiro–Wilk test) were conducted on rock Cu, total Cu, available Cu, and Cu content in tea leaves. The results showed that all data conformed to a normal distribution except for soil available copper. There were no significant differences in rock copper, total copper, and available copper contents in tea garden soils, and copper content in tea leaves among different parent rock types (Table 2). However, metamorphic rocks contained the highest levels of rock copper (14.17 mg/kg) and tea leaf copper (9.24 mg/kg), while sedimentary rocks exhibited the highest total soil copper content (12.60 mg/kg) and magmatic rocks showed the highest available soil copper content (1.46 mg/kg). Except for tea leaf copper, the coefficients of variation for rock copper, total soil copper, and available copper were relatively high across all three rock types.

The average Cu content in the rock of the tea plantations was 14.43 mg/kg (Supplementary Materials S6a). The average total Cu content in the soil of the tea plantations was 12.20 mg/kg (Supplementary Materials S6b), which was well below the screening value (50 mg/kg) for the risk of agricultural land contamination specified in the Soil Environmental Quality Standards [43]. The average available Cu (ACu) content in tea plantation soils was 1.37 mg/kg (Supplementary Materials S7a). The average Cu content in tea leaves in Anxi County was 9.20 mg/kg (Supplementary Materials S7b).

The national [44], Fujian Tieguanyin tea plantation, and Fujian Province [45] soil elemental background values were used as control background values to compare and analyze the average total Cu content in soils derived from different parent rocks in Anxi County (Supplementary Materials S8). The total Cu content of the soils derived from the three major rock types in Anxi County was lower than that of the national and Fujian Province soil elemental backgrounds. Conversely, the total Cu contents of the soils derived from the magmatic, metamorphic, and sedimentary rock backgrounds were higher than that of the soils from the Tieguanyin tea plantation in Fujian Province by 1.05, 1.23, and 1.50 mg/kg, respectively.

#### 3.1.2. Spatial Distribution of Soil and Tea Cu Content

Geostatistical methods were used to fit the sample data to a semivariance function using GS^+^ to select the optimal model. Total soil Cu, ACu, and tea leaf Cu contents in the study area followed the spherical model (Table 3). A residual sum of squares (RSSs) value close to 0 indicates a better fit of the semivariance function model to the data; a nugget effect under 0.25 indicates that the variables have relatively strong spatial autocorrelation, whereas a ratio between 0.25 and 0.75 indicates moderate spatial autocorrelation. The RSS values of total soil Cu, ACu, and tea leaf Cu contents were 2.69, 0.055, and 0.003, respectively, with nugget effects all under 0.25, specifically 0.124, 0.222, and 0.001, respectively. This indicates that the total soil Cu, ACu, and tea leaf Cu contents showed strong spatial autocorrelation.

**Figure 2:**
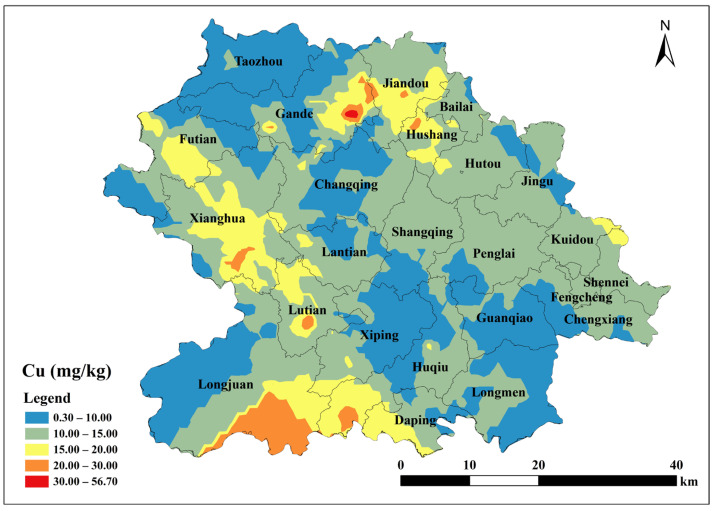
Spatial distribution of Cu content in tea garden soil in Anxi County.

**Figure 3:**
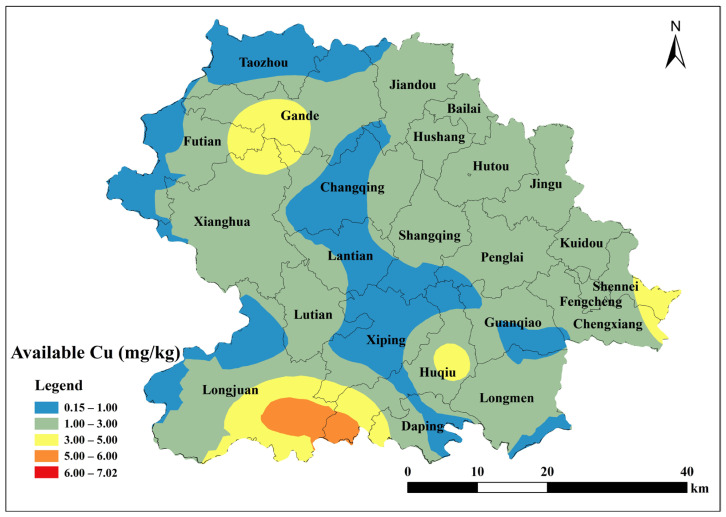
Spatial distribution of available Cu content in tea garden soil of Anxi.

**Figure 4:**
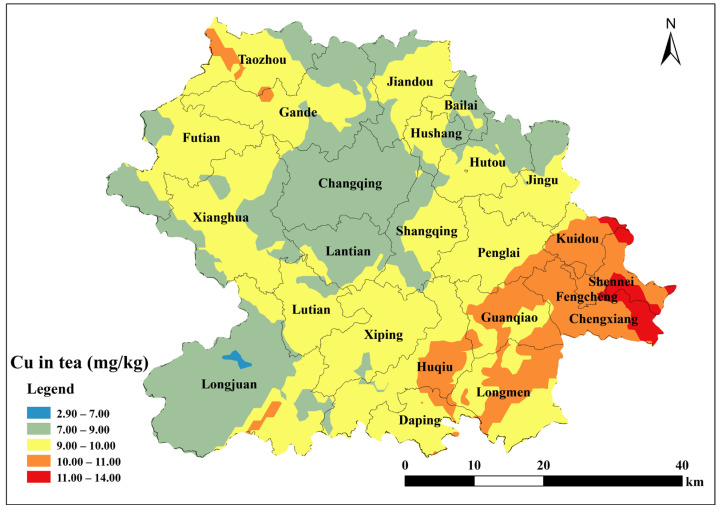
Spatial distribution of tea Cu content in Anxi County.

Based on the parameters of the semivariance function of the spherical model, Kriging interpolation was performed using ArcGIS 10.2 to map the spatial distribution of total soil Cu, ACu, and tea leaf Cu content in Anxi County tea plantations. Total soil Cu content was generally low in the southeast and northwest corners of Anxi County, corresponding to townships such as Longmen, Guanqiao, Huqiu, and Xiping, and high in the north, corresponding to townships such as Sennei, Jiandou, and Lutian (Figure 2). The spatial distribution of ACu content showed higher contents in peripheral townships including Longjuan, Gande, Chengxiang, Sennei, Kuidou, Fengcheng, Hutou, and Jingu, and low in central townships including Changqing, Lantian, Lutian, and Xiping (Figure 3). The spatial distribution of tea leaf Cu content exhibited higher contents in the southeast and northwest and lower contents in the center. Therefore, the highest tea leaf Cu contents were found in the townships of Sennei, Chengxiang, and Kuidou, the second-highest in the townships of Longmen, Guanqiao, and Huqiu, and the lowest in the townships of Longjuan and Changqing (Figure 4).

The total soil Cu content in Anxi County tea plantations ranged from 1.3 to 56.7 mg/kg, with 99.05% of the samples below the Cu risk screening value (50 mg/kg) specified in the Soil Environmental Quality Standards. The enrichment degree of total copper in soil was analyzed with reference to the Specifications for Geochemical Evaluation of Land Quality (DZ/T 0295-2016) [46]. The total Cu content in tea plantation soils of Anxi County was analyzed (Table 4) with soil Cu classified into five grades: Grade I (7.55%), Grade II (2.83%), Grade III (3.77%), Grade IV (8.49%), and Grade V (77.36%). This indicates that only 7.55% of the soils fell into Grade I, whereas the majority (77.36%) were classified as Grade V. Overall, Anxi County tea plantations exhibited low Cu content.

### 3.2. Influencing Factors of Soil and Tea Copper Content

#### 3.2.1. Factors Affecting Soil Copper Content

**Figure 5:**
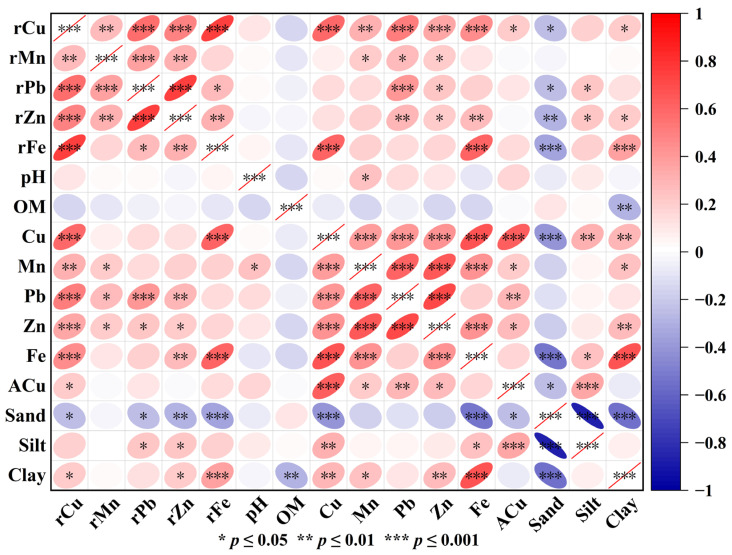
Correlation analysis of rock, soil elements, and soil particles.

Soil heavy metal correlation analysis is commonly used to determine the sources of soil heavy metals, with correlations between elements indicating whether they share the same source. In Anxi County plantations, total soil Cu content exhibited significant correlations with Pb, Zn, and other elements in the soil, parent rock, and soil particles (Figure 5). Total soil Cu showed a highly significant positive correlation (*p* < 0.001) with Cu and Fe in the parent rocks, indicating that soil Cu content was closely related to the Cu and Fe in the parent material. Total soil Cu showed a highly significant positive correlation (*p* < 0.001) with Mn, Pb, Zn, and Fe in the soil, suggesting a companion relationship or a common source for these elements, which is consistent with the findings of Gogoi [4] and Ling [29]. In contrast, total soil Cu showed no correlation with soil pH or soil organic matter, which differs from previous studies. Total soil Cu content was significantly positively correlated with silt and clay content and significantly negatively correlated with sand content, indicating that high sand content significantly inhibited Cu accumulation in soil.

ACu was extremely significantly positively correlated with soil Cu and Pb and significantly positively correlated with rock Cu, soil Zn, and Mn. Conversely, it had no correlation with organic matter and pH (Figure 5). Soil ACu content showed a highly significant positive correlation with silt, a significant negative correlation with sand, and no significant correlation with clay. This pattern, similar to that of total soil Cu, indicates that ACu does not easily accumulate in soils with high sand content.

**Figure 6:**
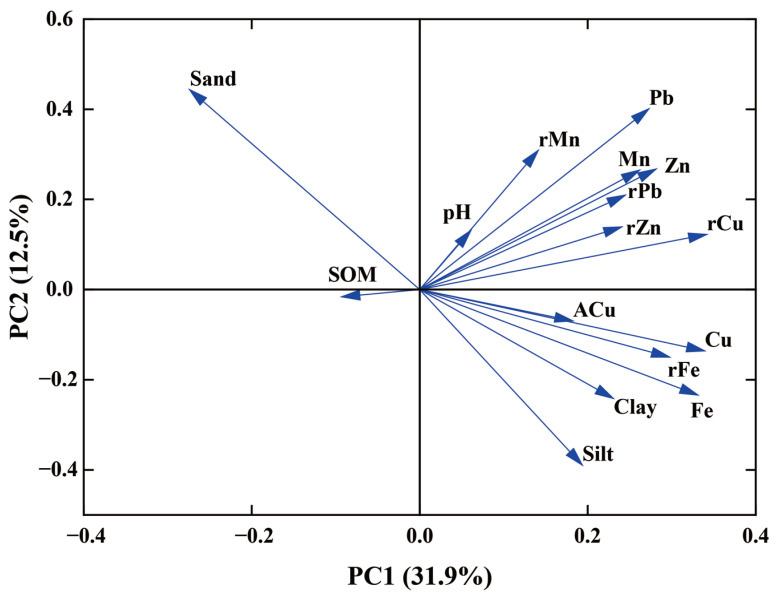
Principal component analysis load diagram (soil Cu).

Principal component analysis was used to identify the sources of Cu in tea plantation soils and determine their contribution to total soil Cu (Figure 6, Table 5). PC1 accounted for 31.9% of the total variance, with parent rock Cu and soil Fe having high loadings, suggesting that these elements are strongly associated with soil Cu and are strongly influenced by the parent rock. PC2 accounted for 12.5% of total variance, with high loadings for soil Pb and sand, indicating that soil Cu and soil Pb share similar sources. PC3 had high loadings for ACu and silt, indicating that biological and chemical transformation of ACu affected soil Cu. In summary, in the analysis of Cu sources in the soil of Tieguanyin tea gardens in Anxi County, PC1 is the parent material-dominated factor, PC2 is the traffic emission factor, and PC3 is the agricultural activity morphological transformation factor.

#### 3.2.2. Factors Affecting Tea Cu Content

**Figure 7:**
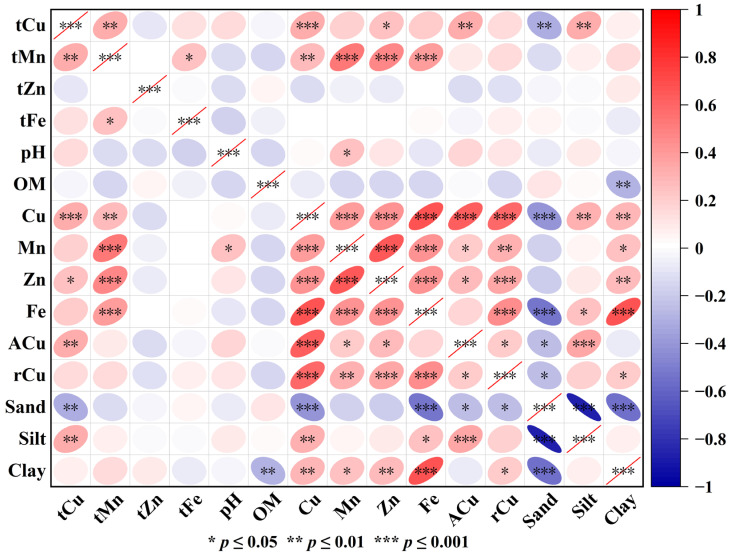
Correlation analysis of soil, tea elements, and soil particles. Among these, tCu, tMn, tZn, and tFe represent the copper, manganese, zinc, and iron content in tea leaves, respectively.

To explore the factors influencing tea leaf Cu content and to understand its relationship with various factors, the Cu content in tea from Anxi County was correlated with other elements in tea, the content of Cu and other elements in the soil, and soil particle composition (Figure 7). Tea leaf Cu content showed a highly significant positive correlation with tea Mn, total soil Cu, and ACu, and a significant positive correlation with soil Mn, Zn, and Fe. It also showed a highly significant negative correlation with sand and a highly significant positive correlation with silt.

**Figure 8:**
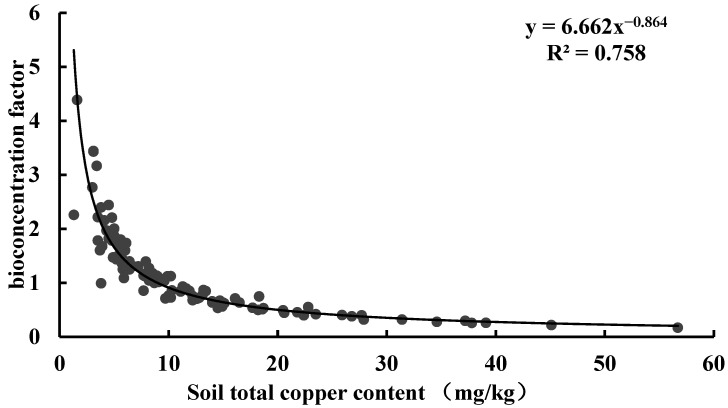
Relationship between bioaccumulation factors and soil Cu content.

**Figure 9:**
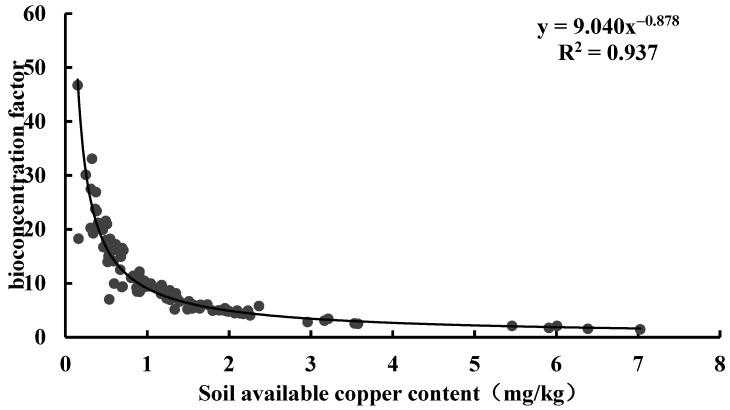
Relationship between bioaccumulation factors and soil available Cu content.

In this study, we evaluated the ability of tea to accumulate Cu using the bioaccumulation factor (BAF). This factor quantifies the ratio of heavy metal content in the plant body to that in the soil and thus assesses the accumulation efficiency of heavy metal transport. A BAF value greater than 1 indicates that the plant can accumulate large amounts of heavy metals from the environment [47]. Based on the ratio of tea leaves to the corresponding soil Cu content in Anxi County, the BAF values were distributed in the range 0.167 to 9.783, with 52.83% of tea samples having BAF values greater than 1, exceeding the random expectation. Based on the ratio of tea leaves to the corresponding soil available Cu content in Anxi County, the BAF values were distributed in the range 1.177 to 75.924, with 100.00% of tea samples having BAF values greater than 1 (Table 6). The highly significant correlation between tea Cu content and soil Cu content confirmed that soil available Cu was the primary source of tea Cu and reflected that tea had a strong enrichment capacity for soil Cu (Figure 7). Curve fitting of the BAF with soil total Cu content revealed a power function relationship between the two, with a coefficient of determination (R^2^) of 0.758 (Figure 8). Further curve fitting of the BAF with soil available Cu content revealed a power function relationship between the two, with a coefficient of determination (R^2^) of 0.937 (Figure 9). This result implies that soil available Cu content is a key factor affecting the BAF in tea.

**Figure 10:**
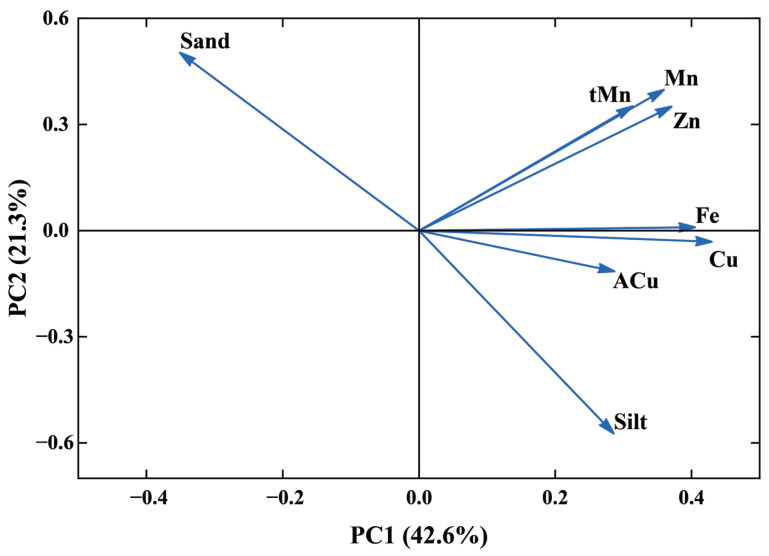
Principal component analysis load diagram (tea Cu).

Principal component analysis was used to identify the sources of Cu in tea and to determine their contributions to tea Cu (Figure 10, Table 7). PC1 accounted for 42.6% of the total variance, with soil Cu, Fe, and Zn having high loadings, suggesting that these elements are strongly correlated with tea Cu and are strongly influenced by soil Cu. PC2 accounted for 21.3% of the total variance, with sand and soil Mn having high loadings, indicating that tea Cu and soil Mn shared similar sources. PC3 showed high loadings for ACu, soil Cu, and sand, suggesting that the biological and chemical transformation process of ACu and soil Cu content influenced tea Cu. In summary, in the analysis of Cu sources in Tieguanyin tea leaves from tea gardens in Anxi County, PC1 is the dominant factor of soil Cu, PC2 is the synergistic factor of soil texture and manganese, and PC3 is the agricultural activity factor.

### 3.3. Assessment of Soil Pollution and Tea Health Risks

#### 3.3.1. Basis for Soil Quality Evaluation

**Figure 11:**
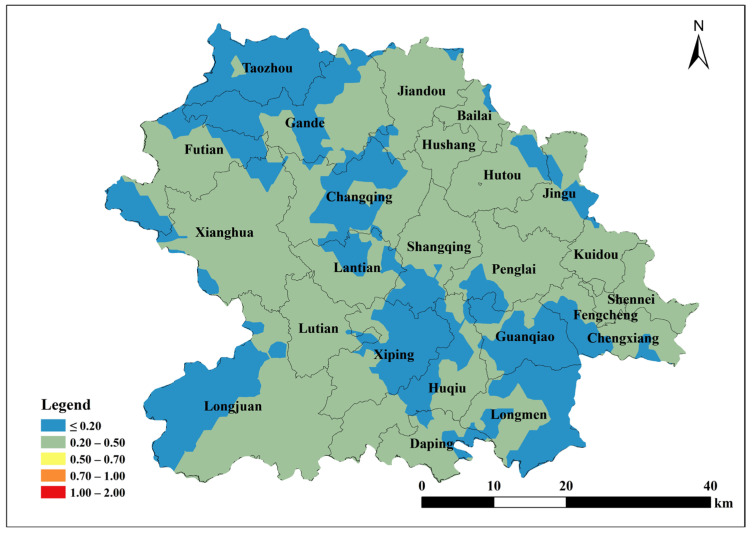
Spatial distribution of single pollution index *P*1 of copper content in tea garden soil in Anxi County.

**Figure 12:**
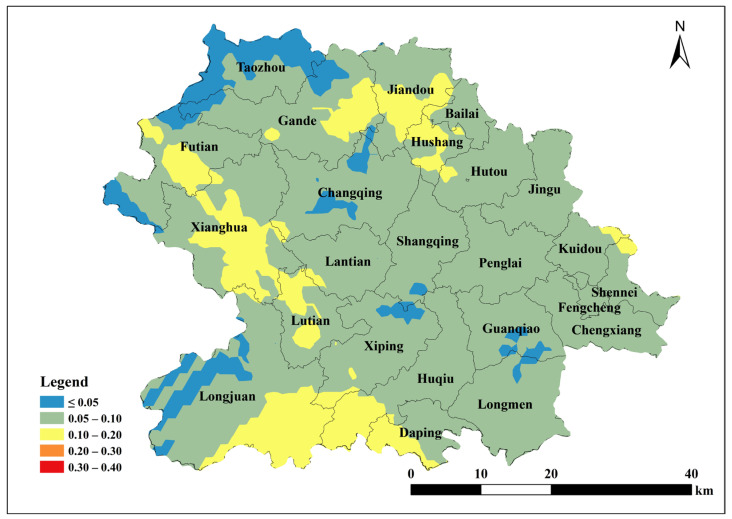
Spatial distribution of single pollution index *P*2 of copper content in tea garden soil in Anxi County.

The single pollution index was calculated using the soil environmental quality standard as the basis for evaluation, combined with the measured soil Cu contents in tea plantations and reference to soil pH (3.07~5.65). Kriging interpolation was performed to obtain the spatial distribution map of this index. Based on the soil environmental quality grade analysis (Table 8), all tea plantation soils in Anxi County were classified as safe (Figure 11). The Soil Environmental Quality Standards categorized only 4.72% of tea plantation soils in Anxi County at an alert pollution level, while the remaining 95.28% remained at a safe level. When using the Environmental Conditions for Harmless Tea Origins as the evaluation standard, the soil of tea plantations in all Anxi County townships were within the safe level (Figure 12), suitable for cultivating and producing pollution-free tea.

#### 3.3.2. Evaluation of Potential Ecological Risks

**Figure 13:**
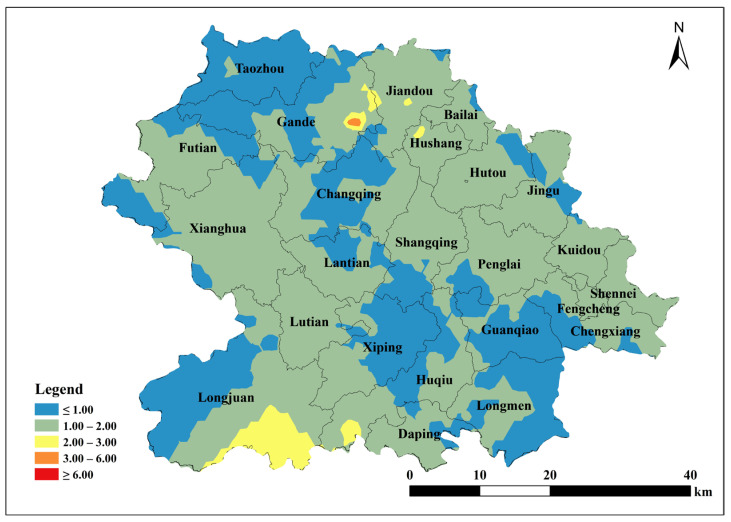
Spatial distribution of potential ecological risk index of total copper content in tea garden soil in Anxi County *E*1.

**Figure 14:**
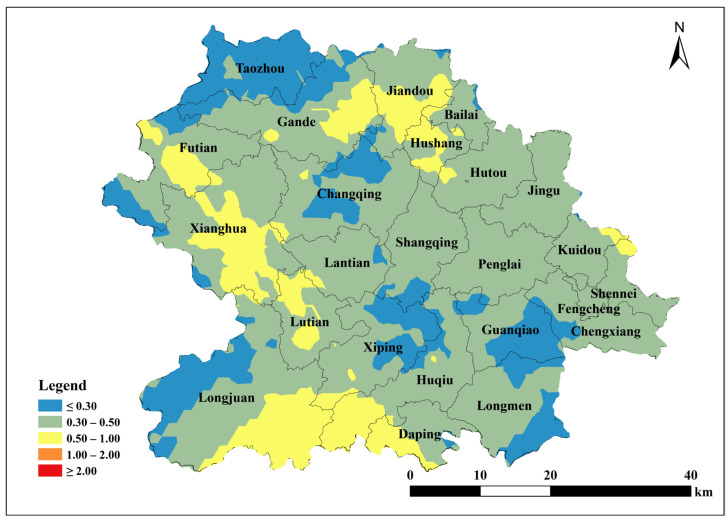
Spatial distribution of potential ecological risk index of total copper content in tea garden soil in Anxi County *E*2.

Using the measured soil copper content and referencing the Cu thresholds in Soil Environmental Quality Agricultural Land Soil Pollution Risk Control Standard (≤50 mg/kg) and Environmental Conditions for Harmless Agricultural Products Tea Origin (≤150 mg/kg), and the toxicity coefficient of Cu (5), the potential ecological hazard index was calculated for soil Cu in the Tieguanyin tea plantation in Anxi County. Evaluations were performed based on different standards: E1 (50 mg/kg) and E2 (150 mg/kg). Kriging interpolation was used to produce spatial distribution maps of the determined potential ecological hazard indices (Figure 13 and Figure 14). The results showed that total soil Cu in the Tieguanyin tea plantation was far below pollution thresholds and posed no potential pollution risk.

#### 3.3.3. Assessment of Tea Health Risks

**Figure 15:**
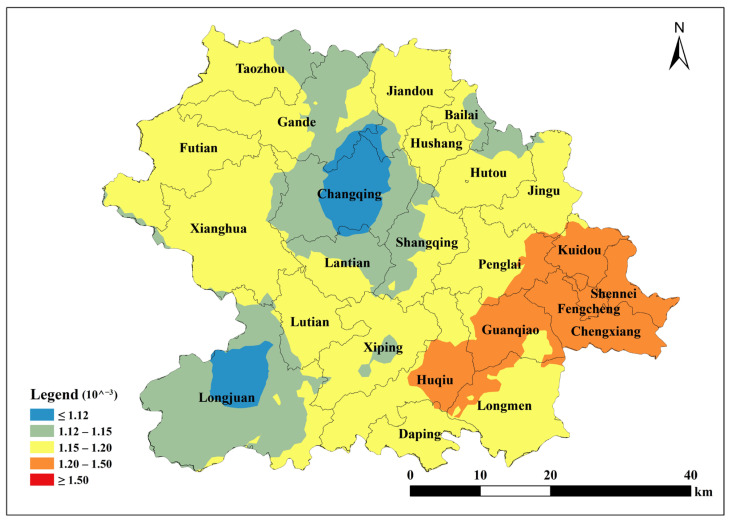
Estimated daily intake of tea Cu in Anxi County.

The Cu contents in all Anxi County tea ranged from 2.94 to 13.68 mg/kg, which were below the standard limits (30 mg/kg) of NY/T 288-2018 and NY 5196-2002, indicating that tea Cu content was within the safety threshold. To further quantify health risks, the *EDI* and *THQ* spatial distributions of tea Cu in Anxi County were determined based on the USEPA health risk assessment model. The *EDI* of tea Cu exhibited a high concentration in the southeast and northwest, with low values in Longjuan, Changqing, Lantian, and other townships (Figure 15). Both *EDI* and *THQ* were less than 1 across all townships, consistent with the report of Sun [48] on the health risk evaluation of soil–tea heavy metals in Anxi Tieguanyin tea plantations. Based on the USEPA health risk assessment, with a *THQ* threshold of 1 for toxicity, Cu content in tea leaves from Anxi County was confirmed to not pose a health risk to humans.

## 4. Discussion

In geology, lithology serves as a key driver of the spatial distribution of soil minerals [49]. This study found that Anxi County primarily develops three types of parent rocks: igneous, metamorphic, and sedimentary. Their differing mineral compositions and weathering characteristics influence copper content in the soil–tea system. Regarding total copper content, soils developed on sedimentary rocks exhibit slightly higher copper levels (12.60 mg/kg), potentially due to trace copper-rich minerals. In contrast, soils developed on igneous rocks show the highest available copper content (1.46 mg/kg), attributed to higher clay proportions and stronger adsorption capacity. Furthermore, significant differences exist in copper content variability among soils developed from the three parent rock types. Igneous rock-derived soils exhibit the highest coefficient of variation (82.44%), reflecting greater spatial variability in their weathering degree.

This indicates that soils developed from metamorphic rocks provide a more stable copper supply, leading to more uniform copper accumulation in tea leaves. In terms of statistical validation of relationships: Pearson correlation analysis reveals that total soil copper is extremely significantly and positively correlated with parent rock Cu and Fe, while tea leaf copper is extremely significantly and positively correlated with soil copper (*p* < 0.01). Spatial overlay analysis further demonstrates that the northern metamorphic rock area corresponds to the high-value zones of soil and tea leaf copper (Gande, Jiandou), while the southern sedimentary rock area corresponds to the low-value zones (Longmen, Guanqiao). The soil Cu content of 85.85% of the samples from the tea plantation soils was lower than that of the natural background value, which is consistent with the conclusion that soil Cu is mainly derived from parent material. Collectively, these results fully confirm that rock type is the core driving factor behind the variations in copper content [50].

The soil Cu content of Anxi County tea plantations showed a highly significant positive correlation with parent rock Cu, parent rock Fe, soil Mn, Pb, Zn, and Fe; a highly significant negative correlation with ACu; a highly significant positive correlation with silt and clay fractions; and a highly significant negative correlation with sand grains (Figure 5). The relationship between total copper content in tea garden soil and soil particles aligns closely with the adsorption–immobilization mechanism of heavy metals by soil particles. Silt and clay particles possess large specific surface areas and surfaces rich in active functional groups such as hydroxyl (-OH) and carboxyl (-COOH) groups. They can immobilize Cu^2+^ through electrostatic adsorption and complexation. Therefore, soils with a high silt/clay fraction are more prone to Cu accumulation. Sand particles have smaller specific surface areas and weaker adsorption capacities. Additionally, soils with high sand content exhibit strong aeration, making Cu more susceptible to migration and loss through leaching. The close relationship between soil copper and metals such as iron, manganese, and zinc is primarily linked to parent rock weathering. Iron is a characteristic element of parent rocks, and its weathering simultaneously releases copper, manganese, and zinc. Examples include minerals like pyroxene (Ca(Mg,Fe,Al)(Si,Al)_2_O_6_) and amphibole found in igneous rocks.

To further investigate the origin of soil Cu, the relevant data were subjected to principal component analysis. The parent rock Cu and soil Fe had higher loadings on PC1, indicating that soil Cu primarily originated from the soil-forming parent material, which is consistent with previous studies [51,52]. Soil Pb and sand exhibited high loadings on PC2, indicating that soil Cu and Pb had similar origins. Traceability analysis revealed that 90.57% of the sample sites had Pb contents exceeding the background values of soil elements in China, indicating that soil Pb is primarily affected by exogenous inputs such as tire wear and exhaust emissions [51,53]. ACu had high loadings on PC3, indicating that the presence of ACu affects soil Cu, which is primarily influenced by agricultural activities such as the application of organic fertilizers. These fertilizers are typically livestock excreta, such as pig manure containing Cu, which is returned to agricultural soil, thereby increasing the soil Cu content [54]. The Cu content of Anxi County tea plantation soils appeared to be higher in the northern regions (i.e., townships such as Gande, Jiandou, and Hushang), which may be related to the application of organic fertilizers.

Tea Cu content showed a highly significant positive correlation with tea Mn, total soil Cu, and ACu; a significant positive correlation with soil Mn, Zn, and Fe; a highly significant negative correlation with sand; and a highly significant positive correlation with silt (Figure 7). Tea trees grow best in soils with a pH of 4 to 6.5; notably, these acidic conditions facilitate the release of metal elements from the soil [55]. Previous studies have found that tea Cu content increases with decreasing soil pH in the eastern Black Sea region of northeastern Turkey [56]. In contrast, no significant relationship between tea Cu content and soil pH was observed in this study, which may be related to the fact that low soil Cu content cannot provide additional Cu sources to tea plants. To further investigate the source of tea Cu, the relevant data were subjected to principal component analysis. Soil Cu, Fe, and Zn had high loadings on PC1, indicating that these elements were strongly associated with tea Cu and strongly influenced by soil Cu. Silt, sand, and soil Mn exhibited high loadings on PC2, indicating that tea Cu and soil Mn originated from similar sources. Tea Cu showed a highly significant positive correlation with tea Mn and a significant correlation with soil Mn, which may be related to the preference of the tea tree for Mn; when soil Mn concentration is high, tea trees absorb and accumulate soil Mn into the plant body [8], and soil Cu can be transferred to tea trees via redox reactions induced by manganese hydroxide [57].

Sand, characterized by large pores and good aeration, accelerates the migration of copper ions within the soil, enabling tea tree roots to more readily absorb them. Their low clay content results in weaker adsorption and immobilization of copper, leading to higher levels of available copper in the soil. Meanwhile, silt particles possess a specific surface area intermediate between sand and clay. They can adsorb copper ions via surface charge, thereby reducing the available copper content in the soil. Additionally, their particle characteristics help buffer soil pH fluctuations, indirectly influencing copper solubility and the efficiency of copper uptake by tea plants. The spatial distribution of tea Cu content in Anxi County was characterized by high concentrations in the southeast and northwest and low, uneven concentrations in the central region. This pattern is inconsistent with the spatial distribution of soil Cu content, indicating that tea Cu content was affected by other factors. Although ACu exhibited a high loading on PC3, the ACu content influenced tea Cu. The available copper content in soil is influenced by fertilization practices, which allows copper to migrate into tea plants and affect their copper levels. This aligns with the spatial distribution observed in this study, where the available copper in Anxi County’s tea garden soils correlates with copper levels in tea leaves.

However, discrepancies exist between areas with high available copper in soil and those with high copper in tea leaves. High available copper zones are primarily distributed in the northern region, while high copper zones in tea leaves are concentrated in the southeast. This indicates that tea leaves absorb copper through additional external pathways. Tracing the sources reveals that besides absorbing copper from the soil, tea plants also absorb it through aboveground pathways such as pesticide spraying. Tea plants are susceptible to diseases like red root rot (*Ganoderma philippii*.), leaf anthracnose (*Colletotrichum* spp.), and dieback (*Fusarium* spp.). To combat these diseases, many farmers spray pesticides containing copper fungicides, such as copper oxychloride and Bordeaux mixture [58,59]. Yaqub et al. [60] found that Zn, Fe, and Cu levels in tea leaves could be influenced by insecticides; therefore, more in-depth mechanistic analysis and validation studies are necessary to reveal whether exogenous aboveground factors are the key drivers of Cu variation in tea.

By combining a single pollution index with a pollution evaluation grading standard for qualitative analysis, the pollution status of the sample sites can be systematically assessed and targeted warnings can be issued accordingly. From the perspective of soil Cu abundance, 7.55% of tea plantation soils were classified as Grade I, 77.36% as Grade V; the spatial distribution was uneven, with most soils exhibiting Cu deficiency. The Cu content of tea plantation soils in each township of Anxi County was evaluated based on the limits of the Soil Environmental Quality Standard and the Environmental Conditions for Pollution-Free Tea Origins. The single pollution index showed that 3.77% of samples were near the critical threshold, while 0.94% were mildly contaminated. The potential ecological hazard indices ranged from 0.03 to 5.67 and 0.01 to 1.89, respectively, both lower than the minimum ecological hazard threshold for single-factor pollutants. Therefore, the Cu content of Anxi County tea plantation soils does not pose a significant ecological risk. Wu et al. [61] reported that human health risks from heavy metal contamination occur primarily through consumption of contaminated crops rather than direct exposure to contaminated soil. Therefore, the potential ecological risk of tea in Anxi County was further assessed using EDI and THQ values, which were in the 10^−3^–10^−2^ range, well below 1, indicating that Cu in Anxi County tea poses no carcinogenic risk to humans.

This study focuses on the distribution, sources, and risks of copper content in soils and tea leaves of Tieguanyin tea gardens in Anxi County. Although phased conclusions have been obtained, there are still limitations. In terms of research region and tea variety, the study is only confined to Tieguanyin tea gardens in Anxi County. The unique geological conditions, climate, and management patterns here are significantly different from those of other tea-growing regions (such as Yunnan and Zhejiang Provinces) and other tea varieties (such as Pu’er tea and Longjing tea), which restricts the generalizability of the conclusions. In terms of research on exogenous influencing factors, it is necessary to distinguish the specific contributions of copper-containing pesticides and organic fertilizers and further quantify the amount of copper input.

Based on the migration characteristics of copper in the soil–tea system of Tieguanyin tea gardens in Anxi County, and combined with the sources and influencing factors of copper, copper accumulation in tea leaves can be reduced through three types of agricultural measures. First, optimize pesticide input: reduce the use of copper-containing pesticides (e.g., Bordeaux mixture) and give priority to biological pesticides such as *Bacillus* preparations and matrine. Meanwhile, apply compound microelement fertilizers containing Zn and Mn (e.g., ZnSO_4_, MnSO_4_). By leveraging the competition between Zn, Mn, and Cu for absorption sites in tea plants, copper uptake by tea plants can be reduced. Second, improve soil physical and chemical properties: for soils with sand content >40%, apply organic amendments such as peat soil and leaf mold to increase the content of soil clay particles and organic matter, thereby enhancing the soil’s capacity to adsorb and fix copper. For acidic soils with pH < 4.5, apply lime to adjust the pH to the range of 4.5–5.5, so as to reduce the bioavailability of copper. Third, optimize tea varieties and planting patterns: in areas with high soil copper content, screen and cultivate superior Tieguanyin varieties with low copper absorption capacity (the copper content in tea leaves of these varieties is 10–15% lower than that of conventional varieties). Additionally, adopt the “tea plant–green manure” intercropping mode (e.g., intercropping with Chinese milk vetch and hairy vetch). Green manure can absorb copper from the soil, and when plowed into the soil, increase soil organic matter content, further regulating the bioavailability of copper in the soil.

Future research can be expanded to multiple tea-growing regions and tea varieties to conduct cross-regional comparative studies, establish long-term fixed monitoring stations to reveal the dynamic changes in copper content, and use gradient experiments to quantify the contributions of exogenous factors. At the same time, a comprehensive pollution risk assessment model should be constructed to expand the assessment dimensions. Eventually, a “region–variety–management measure” trinity strategy for copper control in tea gardens will be formed, providing more comprehensive scientific support for the prevention and control of copper pollution in tea gardens across the country.

## 5. Conclusions

This study evaluated the ecological and health risks of copper (Cu) in soils and tea leaves from Tieguanyin tea plantations in Anxi County. The results showed that the soil Cu content in Anxi tea plantations exhibited significant spatial variability, with obvious differences among tea plantations in different towns. Overall, the soil Cu content in some areas exceeded the soil environmental background value of Fujian Province (21.60 mg/kg), but it was still lower than the limit specified in the Soil Environmental Quality Standard (50.00 mg/kg). This indicates that there is a certain degree of Cu accumulation in the soils of Anxi tea plantations, but not a level constituting serious pollution. Through source identification of Cu, it was found that soil Cu mainly originates from parent materials of soil formation, traffic emissions, and agricultural activities. Soil Cu and agricultural activities are the main sources of Cu in tea leaves. Using the geoaccumulation index method and enrichment factor method for evaluation, it is shown that Cu content in the soil of Anxi tea gardens is far below the pollution level, and there is no potential pollution risk overall. However, in some northern towns such as Cennei, Jiandou, and Lutian, copper content in the soil were close to the limit standard, requiring attention to local control. Combined with the judgment criteria of the health risk assessment model, it was confirmed that the Cu content in tea leaves from Anxi County poses no health risk to humans. In conclusion, this study revealed the distribution characteristics of soil Cu content in Anxi tea plantations, identified the sources of soil Cu and tea Cu through source analysis, and comprehensively assessed the risks of Cu content in soils and tea leaves. It provides a basis for the prevention and control of heavy metals in Anxi tea plantations, so as to construct green and safe tea plantations.

## Figures and Tables

**Table 1:** *EDI* and *THQ* calculation parameters.

Parameter	Implication	Taking Values	Reference
*C*	the content of HMs in tea	2.94~13.68 (mg/kg)	/
*E_F_*	exposed frequency	350 d/a	[35]
*E_D_*	duration of exposure	24 a	[10]
*W_AB_*	average body mass (adult)	60.5 kg	[36]
*T_A_*	average exposure time	ED × 365 d	[10]
*F_IR_*	tea intake rate	8.0 g/d	[37]
*RfD*	oral reference dose	0.04 mg/(kg·d)	[38]

**Table 2:** Rock Cu, soil total Cu, available Cu, and tea Cu content under different rock backgrounds in Anxi County.

	Rock Type	N	Mean Value ± Standard Deviation	Min	Max	Coefficient of Variation
Rock Cu	magmatic rock	88	9.66 ± 15.03 a	0.40	82.30	155.55%
	metamorphic rock	5	14.17 ± 17.23 a	0.80	38.50	121.65%
	sedimentary rock	13	7.36 ± 6.77 a	0.30	23.60	91.98%
Soil total Cu	magmatic rock	88	12.15 ± 10.02 a	1.60	56.7	82.44%
	metamorphic rock	5	12.33 ± 9.59 a	5.50	25.9	77.78%
	sedimentary rock	13	12.60 ± 7.86 a	0.30	27.7	62.34%
Soil available Cu	magmatic rock	88	1.46 ± 0.46 a	0.15	7.02	31.09%
	metamorphic rock	5	1.23 ± 1.00 a	1.10	2.10	81.02%
	sedimentary rock	13	1.39 ± 1.35 a	0.16	3.53	97.52%
Tea Cu	magmatic rock	88	9.24 ± 1.47 a	3.76	13.67	15.94%
	metamorphic rock	5	9.52 ± 0.41 a	9.41	10.38	4.87%
	sedimentary rock	13	8.62 ± 2.08 a	2.94	10.97	24.08%

Note: The letters in the table indicate the differences in copper (Cu) content between different rock types and their developed soils (*p* < 0.05).

**Table 3:** Semivariance function model and corresponding parameters of spatial interpolation of soil copper and tea copper content in tea garden.

Index	Model	Nugget	Sill	Nugget/Sill	R^2^	RSS
total Cu	spherical model	0.206	1.655	0.124	0.134	2.69
available Cu		0.049	0.220	0.222	0.453	0.055
tea Cu		0.0001	0.069	0.001	0.431	0.003

**Table 4:** Cu content richness of tea garden soil.

Cu (mg/kg)	Grading	I	II	III	IV	V
1.3~56.7	range	>29	24~29	21~24	16~21	≤16
	proportion (%)	7.55	2.83	3.77	8.49	77.36

**Table 5:** Factor loading of heavy metal elements in soil.

Element	PC1	PC2	PC3
rCu	0.342	0.122	−0.165
rMn	0.141	0.309	−0.248
rPb	0.244	0.209	−0.454
rZn	0.240	0.139	−0.486
rFe	0.297	−0.150	−0.128
pH	0.061	0.131	0.212
OM	−0.093	−0.016	−0.067
Cu	0.339	−0.136	0.264
Mn	0.261	0.265	0.307
Pb	0.273	0.401	0.169
Zn	0.281	0.267	0.278
Fe	0.331	−0.234	0.050
ACu	0.182	−0.069	0.350
Sand	−0.274	0.443	0.065
Silt	0.193	−0.389	−0.060
Clay	0.230	−0.241	−0.032

**Table 6:** Bioaccumulation factors of Cu in tea.

Variable	BAF Value Range	BAF < 1	BAF > 1
Soil total copper	0.167~9.783	47.17%	52.83%
Soil available copper	1.177~75.924	0.00%	100.00%

**Table 7:** Factor loading of heavy metal elements in tea.

Element	PC1	PC2	PC3
ACu	0.287	−0.115	0.765
Cu	0.430	−0.031	0.399
Sand	−0.351	0.503	0.325
Zn	0.371	0.350	−0.074
Mn	0.359	0.398	−0.100
Silt	0.286	−0.573	−0.151
tMn	0.313	0.351	−0.232
Fe	0.405	0.010	−0.242

**Table 8:** Soil environmental quality grade.

Grade	Pollution Index	Class of Pollution
1	*P* ≤ 0.7	Safety
2	0.7 < *P* ≤ 1.0	Warning line
3	1.0 < *P* ≤ 2.0	Mild pollution
4	2.0 < *P* ≤ 3.0	Moderate pollution
5	*P* > 3.0	Heavy pollution

## Data Availability

The data presented in this study are available upon reasonable request from the corresponding author.

## Supplementary Materials

The following supporting information can be downloaded at: https://www.mdpi.com/article/10.3390/toxics13121042/s1, Supplementary Material S1. Descriptive Statistical Analysis of Soil Physicochemical Properties and Heavy Metal Content in Rocks and Soil. Supplementary Material S2. The quality assurance/quality control (QA/QC) procedures for all analytical testing. Supplementary Material S3. Classification of ecological hazard degree and toxicity coefficient of single factor pollutants. Supplementary Material S4. Pollution limits of Cu in tea garden soil under different standards. Supplementary Material S5. Cu pollution limits of tea under different standards. Supplementary Material S6. (a) Frequency distribution of rock Cu. (b) Frequency distribution of soil total Cu. Supplementary Material S7. (a) Frequency distribution of soil available Cu content. (b) Frequency distribution map of Cu content in tea. Supplementary Material S8. Comparison of total copper content and background value of soils with different rock development in Anxi County.
