# Supplementary material for: Distribution and Risk Assessment of Copper Content in Soil and Tea of Tieguanyin Plantations in Anxi County, China
Source: Toxics. 2025 Nov 30;13(12):1042. doi: 10.3390/toxics13121042 (PMC12737622; doi:10.3390/toxics13121042)
Supplement: Supplementary file 1 [file toxics-13-01042-s001.zip › toxics-3986817-supplementary.pdf]

Supplementary Material **S1**: Descriptive Statistical Analysis of Soil Physicochemical Properties and Heavy Metal Content in Rocks and Soil

**Indicator**	**Mean Value**	**Standard Deviation**	**Min**	**Max**	**Coefficient of Variation**
Rock Mn	737.88	1156.71	78.00	9741.00	156.76%
Rock Pb	51.89	83.81	8.10	640.60	161.53%
Rock Zn	73.00	81.01	8.90	572.90	110.98%
Rock Fe	23890.68	23978.73	3217.24	182473.46	100.37%
Soil Mn	397.71	366.24	65.00	2356.00	92.09%
Soil Pb	57.42	75.81	14.90	700.60	132.04%
Soil Zn	71.68	36.43	20.90	262.80	50.81%
Soil Fe	34207.18	16815.48	11819.86	106308.80	49.16%
pH	4.29	0.44	3.07	5.65	10.22%
OM	26.94	10.62	0.04	72.62	39.43%
Sand	30.58	14.51	5.38	90.12	47.46%
Silt	59.16	12.20	9.56	80.75	20.62%
Clay	10.26	7.26	0.31	29.55	70.73%

Supplementary Material **S2**: The quality assurance/quality control (QA/QC) procedures for all analytical testing

To ensure the accuracy and reliability of detection data, all experiments strictly adhere to the following quality assurance/quality control measures: First is instrument calibration and verification. Before using the ICP-MS 2000E, calibrate it with 10 μg/L and 100 μg/L multi-element standard solutions (containing target elements such as Cu, Mn, Zn, and Pb) to ensure that the instrument response deviation ≤5%. After every 20 sample determinations, perform an intermediate check using a 50 μg/L standard solution; recalibrate if deviation exceeds the limits. Before using the laser particle size analyzer (BT-9300ST), verify it with 10 μm and 100 μm standard latex particles, ensuring a measurement deviation ≤2%. Calibrate the pH meter (FE28-Standard) with pH 4.00 and 6.86 standard buffers before each experiment. After calibration, measure the pH of the blank solution with an error ≤±0.05. Second is batch quality control for samples. For each batch, set up two reagent blanks (digestion reagent only, no sample), two parallel samples (repeated digestion and measurement of the same sample), and one Standard Reference Material (SRM). Soil samples use the "Standard Reference Materials for Soil Component Analysis" (GBW07405, Cu standard value 25.8±1.5 mg/kg), whereas tea samples use the "Standard Reference Materials for Tea Component Analysis" (GBW10052, Cu standard value 10.2±0.8 mg/kg). Blank control requirements are as follows: target elements (Cu, Mn, Zn, etc.) must not be detected; the relative deviation between parallel samples must be ≤5%, retest if exceeded; and the relative error between measured and certified values of SRM must be ≤8% to ensure method accuracy. Finally, data validity control is performed. All raw data undergo Grubbs' test for outliers (significance level α=0.05). Detected outlier samples require redissolution and retesting; data may be included in statistics only after confirmation of accuracy. Results for soil available Cu, total Cu, and tea Cu must satisfy the following: Method Detection Limit (LOD) < Result < 1000 times the Method Quantification Limit (LOQ). If values fall outside this range, adjust the sample weight or dilution factor and retest. All reagents used in the experiment (nitric acid, hydrofluoric acid, etc.) were of analytical grade purity. Experimental water was ultrapure water (resistivity ≥18.2 MΩ · cm). All glassware and PTFE digestion vessels were immersed in 10% nitric acid for 24 hours, rinsed three times with ultrapure water, and dried before use to prevent cross-contamination.

Supplementary Material **S3：**

Classification of ecological hazard degree and toxicity coefficient of single factor pollutants.

**Ecological Hazard Degree of Single Factor Pollutants**	**Low**	**Medium**	**Relatively High**	**High**	**Extremely High**	**Toxicity Coefficient (Cu)**
E	＜40	40-80	80-160	160-320	≥320	5

Supplementary Material **S4：**

Pollution limits of Cu in tea garden soil under different standards.

**Standard**	**Range(mg/kg)**
《Soil environmental quality standard》 (GB 15618-2018)	≦50
《Environmental conditions of pollution-free tea producing area》（NY5020-2001）	≦150

Supplementary Material **S5**：

Cu pollution limits of tea under different standards.

Standard	Range(mg/kg)
《Green food tea》（NY/T 288-2018）	≦30
《Organic tea》（NY 5196-2002）	≦30

Supplementary Material **S6：**

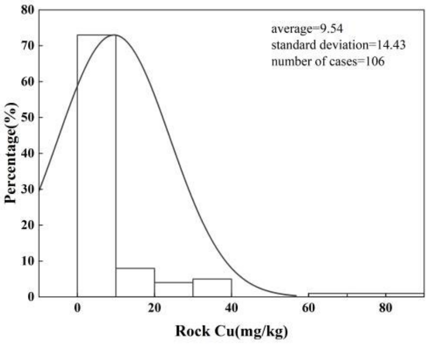

(**a**)

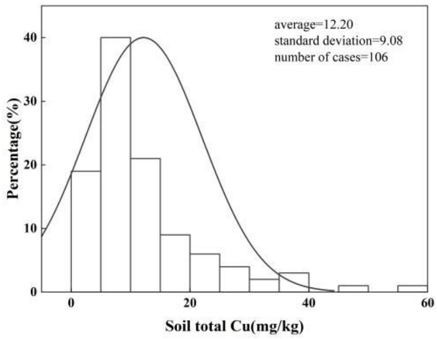

(**b**)

(**a**) Frequency distribution of rock Cu. (**b**) Frequency distribution of soil total Cu.

Supplementary Material **S7：**

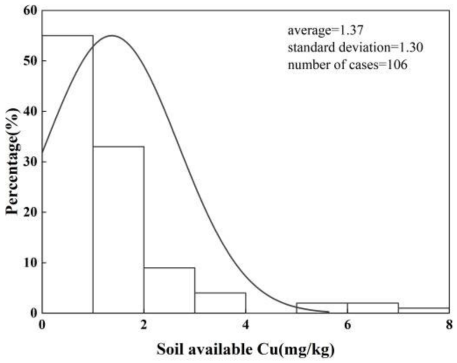

(**a**)

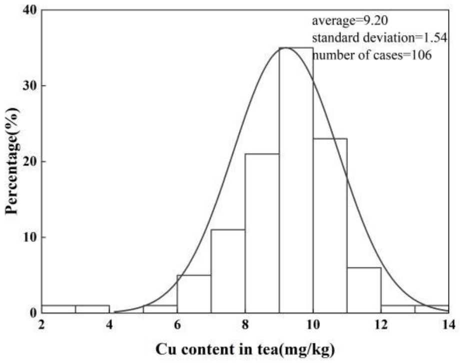

(**b**)

(**a**) Frequency distribution of soil available Cu content. (**b**) Frequency distribution map of Cu content in tea.

Supplementary Material **S8:**

Comparison of total copper content and background value of soils with different rock development in Anxi County.

	Rock Type	A	B	C	D	AB	AC	AD
three major rocks	magmatic rock	12.15	18.20	11.10	21.60	-6.05	1.05	-9.45
	metamorphic rock	12.33	18.20	11.10	21.60	-5.87	1.23	-9.27
	sedimentary rock	12.60	18.20	11.10	21.60	-5.60	1.50	-9.00

Note: A represents the average copper content of different rock development soils in Anxi County; B, C, and D represent the background value of Cu in Chinese soil, the background value of Cu in Tieguanyin tea garden soil, and the background value of Cu in Fujian soil, respectively. AB, AC, and AD represent the difference between the mean value of copper content and the background value of different rock development soils in Anxi County, respectively.
